# Non-spherical gold nanoparticles enhanced fluorescence of carbon dots for norovirus-like particles detection

**DOI:** 10.1186/s13036-023-00351-x

**Published:** 2023-04-27

**Authors:** Abdulhakeem Alzahrani, Tawfiq Alsulami, Ahmad Mohammad Salamatullah, Syed Rahin Ahmed

**Affiliations:** 1 Department of Food Science & Nutrition, College of Food and Agricultural Sciences, King Saud University, Riyadh, 11451 Saudi Arabia; 2 School of Engineering Practice and Technology, McMaster University, 1280 Main Street West, Hamilton, ON L8S 4L8 Canada

**Keywords:** Carbon dots, Gold nanoparticles, Norovirus-like particles, Exciton-plasmon interaction, Fluorescence sensor

## Abstract

**Background:**

Norovirus is a common pathogen that causes foodborne outbreaks every year and the increasing number of deaths caused by it has become a substantial concern in both developed and underdeveloped countries. To date, no vaccines or drugs are able to control the outbreak, highlighting the importance of finding specific, and sensitive detection tools for the viral pathogen. Current diagnostic tests are limited to public health laboratories and/or clinical laboratories and are time-consuming. Hence, a rapid and on-site monitoring strategy for this disease is urgently needed to control, prevent and raise awareness among the general public.

**Results:**

The present study focuses on a nanohybridization technique to build a higher sensitivity and faster detection response to norovirus-like particles (NLPs). Firstly, the wet chemical-based green synthesis of fluorescent carbon quantum dots and gold nanoparticles (Au NPs) has been reported. Then, a series of characterization studies were conducted on the synthesized carbon dots and Au NPs, for example, high-resolution transmission emission microscopy, fluorescence spectroscopy, fluorescence life-lime measurement, UV–visible spectroscopy, and X-ray diffraction (XRD). The fluorescence emission of the as-synthesized carbon dots and the absorption of Au NPs were located at 440 nm and 590 nm, respectively. Then, the plasmonic properties of Au NPs were utilized to enhance the fluorescence emission of carbon dots in the presence of NLPs in human serum. Here, the enhanced fluorescence response was linearly correlated up to 1 μg mL^−1^. A limit of detection (LOD) value was calculated to be 80.3 pg mL^−1^ demonstrating that the sensitivity of the proposed study is 10 times greater than that of the commercial diagnostic kits.

**Conclusions:**

The proposed exciton-plasmon interaction-based NLPs-sensing strategy was highly sensitive, specific, and suitable for controlling upcoming outbreaks. Most importantly, the overall finding in the article will take the technology a step further to applicable point-of-care (POC) devices.

**Supplementary Information:**

The online version contains supplementary material available at 10.1186/s13036-023-00351-x.

## Background

Norovirus, a foodborne pathogen, is the main cause of epidemic gastrointestinal diseases. Norovirus is extremely infectious, and outbreaks usually occur in the winter seasons. The infection initiated by this virus is generally spread through contact between person-to-person and is initiated by contaminated food, water, and surfaces. The most common symptoms of this infectious virus are vomiting and diarrhea which result in approximately 220,000 yearly deaths, of which 70% are children under five years old. Hence, a rapid and sensitive detection technique is crucial to identifying the presence of norovirus and preventing food-induced harm [1–3]. Recently, a non-infectious form of the virus (known as virus-like particles) has been gaining research interest because it does not contain any viral genetic material and is safe to handle without extra precaution.

The traditional cell culture-based detection and the reverse transcription polymerase chain reaction for norovirus detection are extremely complex which prevents their use in onsite rapid detection. Immunoassay-based techniques are comparatively simpler, making them the more favoured option. Though the colorimetric immunoassay technique is very simple, it takes a slightly longer detection time and provides low sensitivity. The fluorescence immunoassay technique is well-known for providing a much faster detection response, is specific to target analytes and has onsite applicability.

Over the last several years, nanomaterials have received a lot of research focus because of their tremendous impact on biology, medicine, and pharmaceutical sciences [4–15]. In particular, the application of fluorescent nanostructures in different fields is increasing dramatically and the development of new synthesis methods of different fluorescent nanostructures is earning popularity [16–20]. Carbon-based nanomaterials are intensively studied in the field of modern nanoscience as an alternative to heavy metal-containing fluorescent semiconductor nanocrystals due to their excellent photostability, biocompatibility, and low toxicity. They also unveil new possibilities as a metamaterial in catalysis and biosensing applications for advanced optical devices [21–30].

To date, there are several different synthesis methods of carbon dots that have been reported, for example, the hydrothermal method, laser ablation technique, arc discharge technique, electrochemical approach, microwave irradiation method and direct pyrolysis approach [31, 32]. Among different synthesis approaches for carbon dots, the hydrothermal method is most frequently employed because it provides an easy, clean and efficient way. Moreover, carbon sources from renewable and waste materials are economical, environmentally friendly and ideal candidates for the green synthesis of carbon dots. Tan and co-workers have synthesized carbon dots from sago industrial waste using a thermal pyrolysis approach [33]. The article has reported that the size and optical properties of the carbon dots can be tuned by varying the temperature and exhibited a novel approach to using agricultural waste in optical sensing receptors. Raveendran et al., have reported the synthesis of highly luminescent carbon dots from the mint leaf through hydrothermal treatment [34]. The size of the synthesized carbon dots was 4 nm with hydroxyl groups on their surface. The emission properties of carbon dots were excitation-dependent and stable in a range of media. The hydrothermal carbonization synthesis method of carbon dots from orange peel waste has been prepared by Prasannan and his co-worker [35]. The synthesized carbon dots were applied for dye degradation and results showed great potential for catalytic performance in biowaste. An impressive synthesis of carbon dots using the Pseudo-stem of banana plants has been reported by Vandarkuzhali and co-workers [36]. A quantum yield of approximately 48% was achieved through this method. The photostability and biocompatibility of synthesized carbon dots were satisfactory. However, there are several drawbacks to these reported articles, for example, it requires a higher temperature for synthesis and produces larger-sized carbon dots. Hence, a green synthesis procedure of carbon dots is needed that enables to prepare smaller-sized carbon dots in shorter reaction time.

Due to their unique properties, carbon dots have been applied in diverse fields. To date, several research articles have reported on the fluorescence-based biosensing applications of carbon dots. Most of these sensing methods rely on the fluorescence quenching of carbon dots in the presence of target analytes. The possibility of fluorescence enhancement in close proximity to plasmonic surfaces is less explored which might be a promising route in biosensing applications. To fill this gap, the present study aims to synthesize carbon dots from a green source and then modify the optical properties of carbon dots using surface plasmon properties of plasmonic nanoparticles. In particular, the selection of green sources to produce carbon dots will be advantageous for building an ecological community since it is environmentally friendly and helps in reducing waste production. Citrus macroptera is a well-known fruit in South Asian countries and is used in making different curries. The pulp of citrus macroptera is usually dumped because of its unpleasant taste. In this study, we aimed to use the pulp of citrus macroptera to synthesize carbon dots through a hydrothermal approach.

Au NPs demonstrate superior surface-plasmon resonance properties that exhibit distinct optical properties and solution color depending on particle size. To date, a huge number of raw materials have been used to synthesize Au NPs, indicating that the synthesis of Au NPs is comparatively easy. The size and shape of Au NPs can be easily tuned by varying concentrations of reducing agents, this has a tremendous impact in several scenarios. Along with other advantages, the ease of bioconjugation of Au NPs with biomolecules (antibodies, aptamers) and long-time stability make it a unique and promising candidate for biosensing applications. Though a large volume of studies regarding Au NPs in biosensing applications has been reported based on their optical nature, colorimetric behavior and nanozymatic activity [37–42], only a few articles have reported the influence of Au NPs shape in enhancing its detection sensitivity. More research should be done in this area to know the possibility of the fabrication of non-spherically shaped nanomaterials and their applications in different areas. It is well-known that rough plasmonic surfaces show better optical scattering compared to smooth surfaces and helps to increase the sensitivity of detection.

The advancement of the nanohybridization technique allows us to modify the properties of nanomaterials which is promising in enhancing the sensitivity of the biosensor. For example, nanohybridization of quantum dots (QDs) and plasmonic nanoparticles might change the emission of quantum dots, enhance the absorption of plasmon nanoparticles, and modify the fluorescence decay time due to the exciton and plasmon interaction [12, 43, 44]. Importantly, the modification of optical phenomena of QDs strongly relies on the surface morphology of the plasmonic nanostructure which enables unique control over optical properties and plays a vital role in near-field optics-based applications. In general, the plasmonic rough surface contains higher defect sites, distinct electronic states, and strong absorption nature. These unique features help in the modification of the optical response of QDs through coupling plasmonic absorption of metallic nanostructures and excitonic energy of QDs. For example, a six-fold enhancement of fluorescence emission and drastic reduction of fluorescence decay properties of CdSe/ZnS QDs on non-homogeneous Ag nanoneedle surfaces compared to smooth surfaces have been reported. Here, the optical scattering of the plasmonic rough surface plays a significant role in the fluorescence enhancement of QDs [44]. Those optical changes are crucial to building ultra-sensitive nanobiosensors in the presence of target analytes [41].

In this study, we aimed to utilize the exciton-plasmon coupling of carbon dots and the rough plasmonic surface of Au NPs to develop a highly sensitive biosensor to detect NLPs in human serum. Firstly, new synthesis methods for both carbon dots and Au NPs had been developed. Next, the synthesized carbon dots and Au NPs were bound with target-specific antibodies and brought at a nanoscale distance in the presence of NLPs (Scheme S1). Then, the changes in fluorescence emission were monitored and enabled the quantification of the target analyte spectroscopically.

## Results

**Scheme 1:**
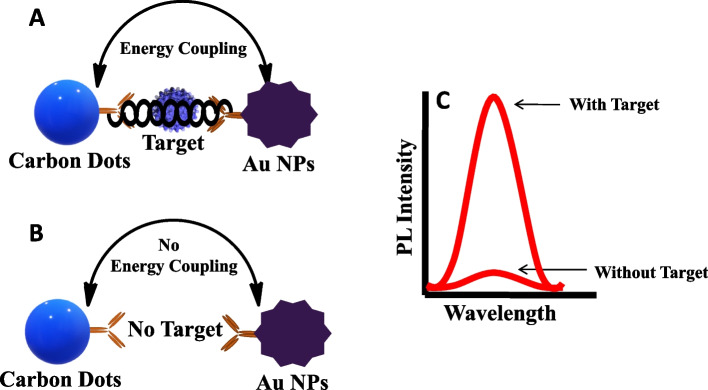
Schematic presentation of NLP detection

In this study, the exciton-plasmon coupling was utilized to develop a sensitive and rapid NLP detection. It is well-known that excitonic properties of quantum dots and surface plasmon properties of plasmonic nanomaterials interact with each other’s at the close proximity. Such interaction modifies the emission properties of quantum dots. In particular, rough plasmonic surface plays a significant role in enhancing the emission properties of quantum dots. Here, we aimed to utilize this strategy for NLPs detection. As presented schematically, antibody-conjugated Au NPs and antibody-conjugated carbon dots interacted with each other and came within nanoscale distance in the presence of the target virus (Scheme 1). At this stage, plasmonic and excitonic properties of Au NPs and carbon dots brought the system to a higher energy regime, and any small changes affected the fluorescence significantly. For example, the changes in target virus concentration affected the fluorescence emission and helped to detect it at a low concentration. Without the target virus, antibody-conjugated Au NPs and antibody-conjugated carbon dots did not show any interaction, and the fluorescence emission did not change.

**Fig. 1:**
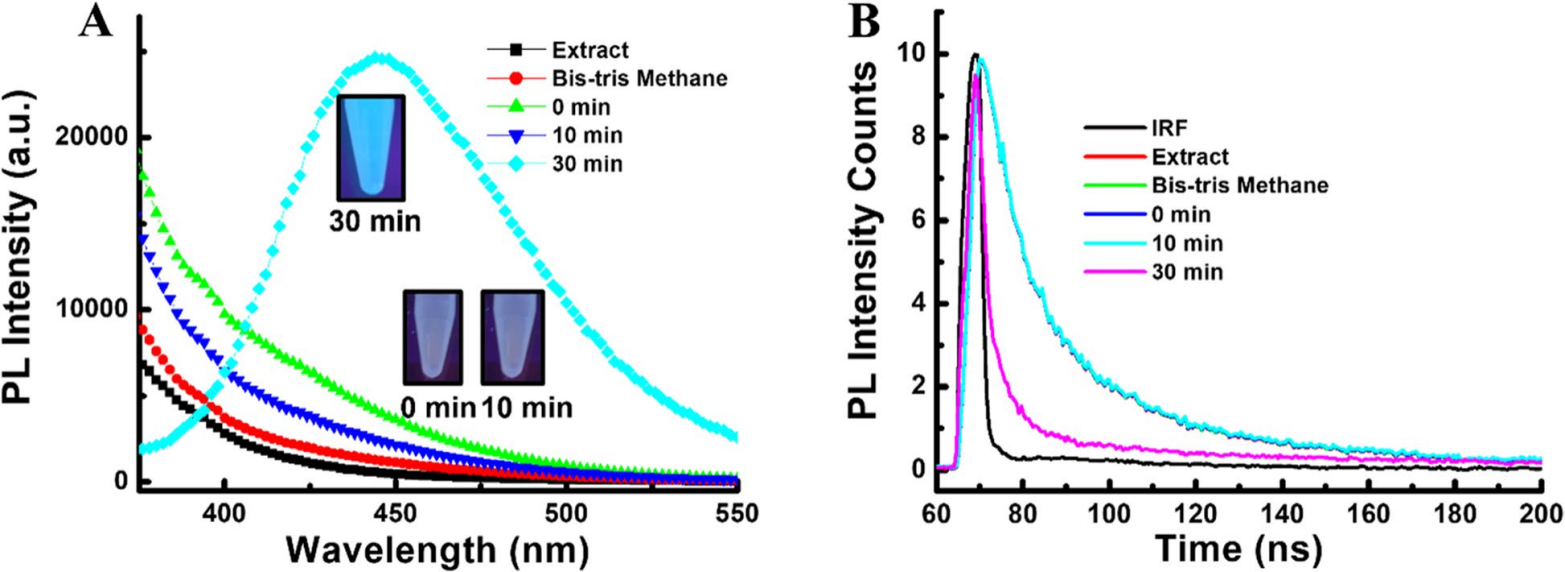
Characterization of carbon dots: (**A**) Photoluminescence (inset: photograph of the solution under UV-light irradiation) & (**B**) Time-dependent PL decay profile during synthesis of carbon dots

At first, the photoluminescence (PL) spectrum of synthesized carbon dots was measured, which showed a strong PL emission peak centered at 440 nm when excited at 320 nm (Fig. 1A). It takes around 30 min for the fluorescence emission properties in carbon dots to appear. The fluorescent photography of the solution under UV light irradiation revealed that a minimum of 30 min of hydrothermal reaction time is required to produce carbon dots. Moreover, the PL decay rate of the solution was measured using a 380-nm excitation wavelength from a light-emitting diode (LED; PTI Inc., Oakland, CA, USA). As shown in Fig. 1B, the PL profile of carbon dots (30 min reaction time) decreased significantly compared to the samples at 0 min and 10 min reaction time. The measured decay of carbon dots was 1.96 ns. These observations appeared because of the higher number of surface defects of the carbon dots that fed the non-radiative recombination process. EDX data of synthesized carbon dots revealed the presence of carbon in solution (Fig. S1) with a negative charge (-15.9 eV) on the surface because of the –OH groups (Fig. S2).

The crystallinity of fluorescent carbon dots was investigated using X-ray diffraction (XRD) analysis. The diffraction peak is located at 26.4º corresponding to the interlayer spacing of the graphite structure (0.34 nm interlayer spacing) for the lattice fringes of (002) planes (Fig S3) [45].

**Fig. 2:**
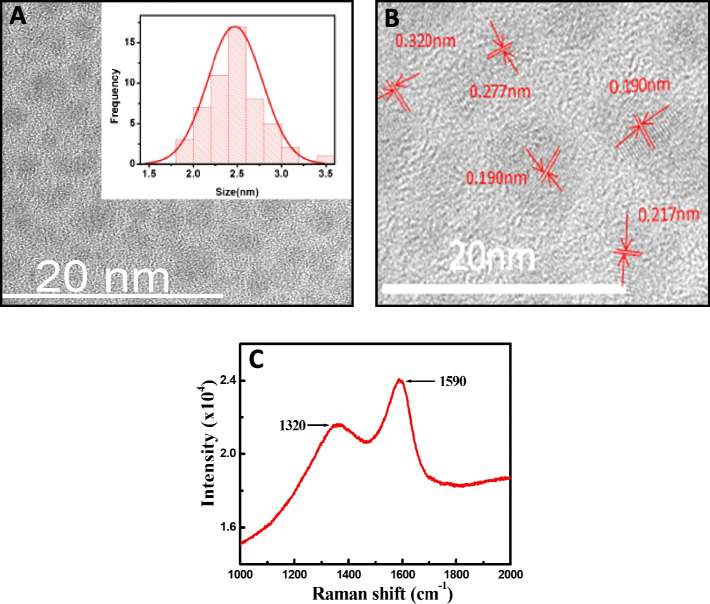
HRTEM images of carbon dots: (**A**) HRTEM image of monodispersed carbon nanomaterials (inset: bar chart of nanomaterials size); (**B**) Crystalline nature of the carbon dots. (**C**) Raman spectroscopy of carbon dots

The formation and particle core diameter of carbon dots were confirmed by HRTEM measurements. The HRTEM micrograph of the carbon nanoparticles is shown in Fig. 2A, and it is seen that the particles are quasi-spherical in shape with a nearly monodisperse size distribution (2.5 ± 1 nm). In addition, the HRTEM image shows the crystalline nature of the carbon dots in Fig. 2B. The observed d-spacings are 0.190 nm, which may be attributable both to the (105) diffraction plane of diamond-like (sp^3^) carbon and to the (105) lattice of graphitic (sp^2^) carbon, whereas further analysis indicates that the carbon dots obtained are of graphitic structure because several other lattice fringes were found with the lattice spacings of 0.32 nm, 0.277 nm, and 0.217 nm, which are close to the (006), (020) and (101) diffraction facets of graphite carbon, respectively [46, 47].

The chemical structure of carbon dots was measured using Raman spectroscopy and is shown in Fig. 2C. The spectrum contains two major peaks, a diamond peak of carbon located at ~ 1320 cm^−1^ that appeared due to sp^3^ nature, and a sharp upward band peak centered around ~ 1590 cm^−1^ representing the G-band of sp^2^ graphitic nature of carbon materials [45]. A ratio of the intensity (ID/IG) of the carbon plate was ca. 0.79, indicating that the graphitic composition of carbon dots had high purity.

**Fig. 3:**
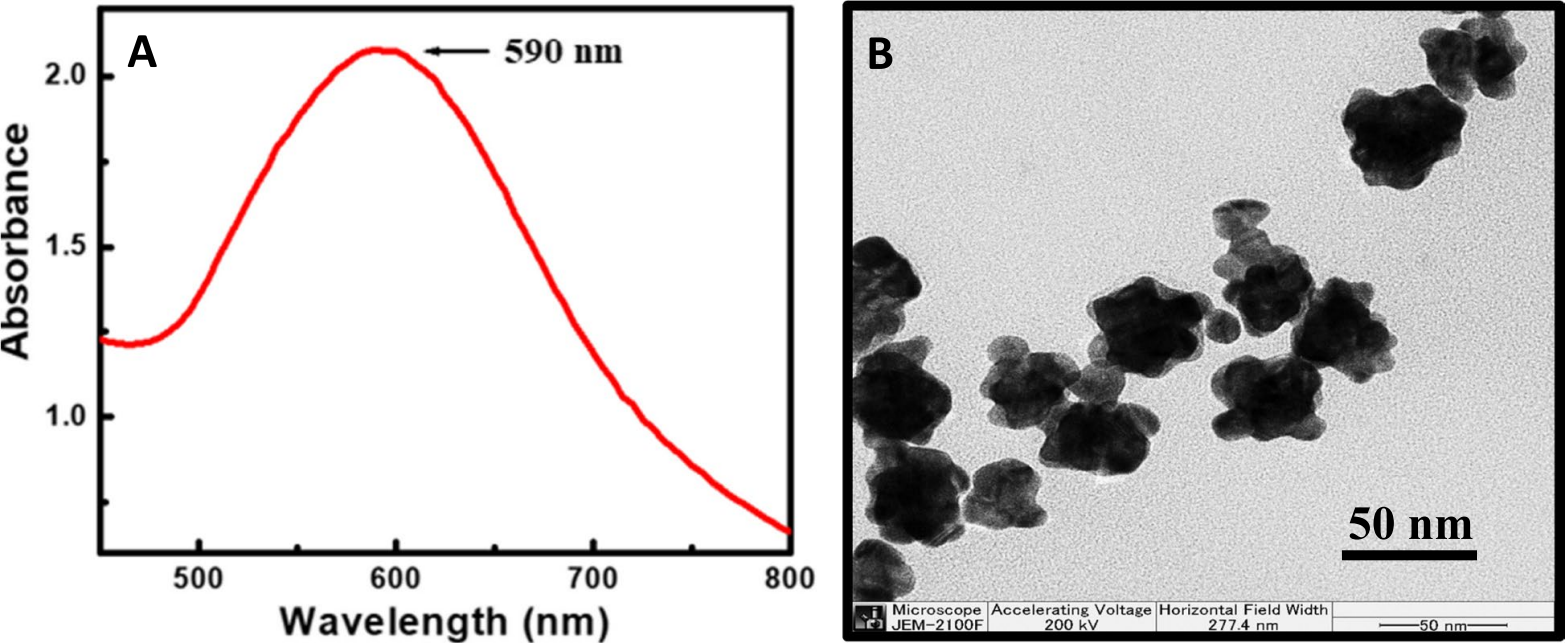
Characterization of Au NPs: (**A**) absorbance and (**B**) TEM image of Au NPs

The absorbance peak of synthesized Au NPs was located at 590 nm (Fig. 3A). TEM images revealed that Au NPs shape was non-spherical, and the size of approximately 50 nm (Fig. 3B). The non-spherical shaped Au NPs can modify optical properties through plasmonic scattering during exciton-plasmon coupling and enable the improvement of the detection sensitivity of a biosensor. Both nanomaterials (carbon dots and Au NPs) showed well stability up to 6 months (Fig. S4).

**Fig. 4:**
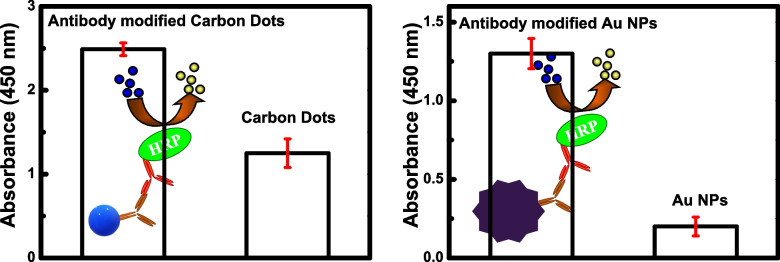
ELISA results of binding confirmation: (Left) carbon dots with antibodies and (Right) Au NPs with antibodies

Before the start of NLP sensing experiment, ELISA was performed to confirm the binding of antibodies with carbon dots and Au NPs. In this study, antibodies were prepared in-solution at a pH of 5 to get a positive net charge (Fig S5A). This facilitated electrostatic binding with negatively charged carbon dots (Fig S5B). As shown in Fig. 4, a higher absorbance intensity was achieved with antibody-bound carbon dots (Fig. 4A) and antibodies-bound Au NPs (Fig. 4B) samples in comparison to the bare nanomaterials, revealing that antibodies are tightly bound with both nanomaterials.

**Fig. 5:**
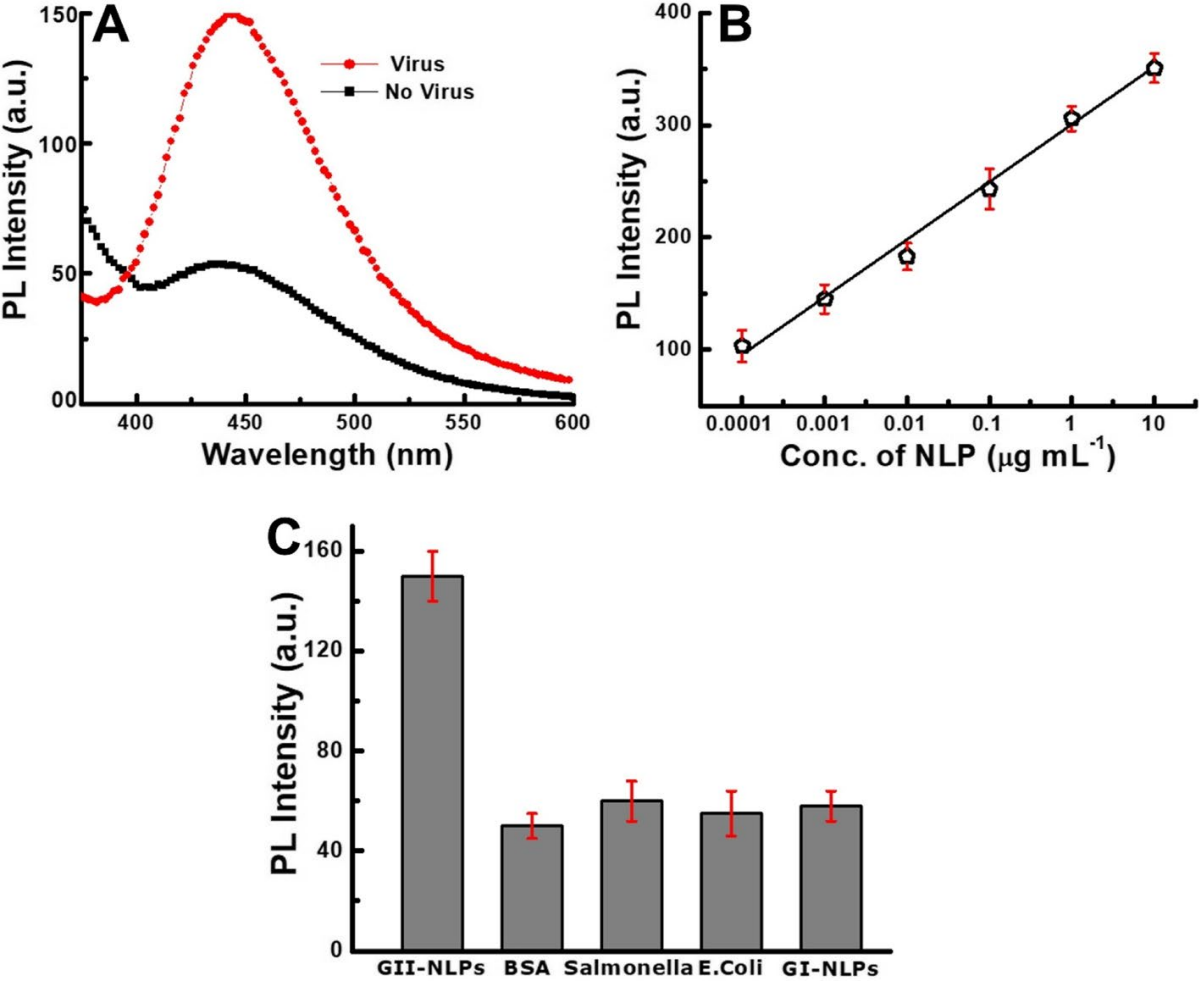
Detection of NLP: (**A**) control experiment; (**B**) calibration curve of fluorescence Vs different concentrated NLP; (**C**) The specificity of the proposed method. Here, the concentration of BSA, *Salmonella*, *E. Coli,* and GI-NLPs was 0.0012 µg mL^−1^, 0.0011 µg mL^−1^, 0.001 µg mL^−1^ and 0.001 µg mL^−1^, respectively

After confirming the binding of nanomaterials with the antibodies, the sensing experiments were performed. At first, a control experiment was examined to check the influence of the target virus on the fluorescence emission properties of carbon dots. As shown in Fig. 5A, the emission had enhanced significantly in the presence of the virus (0.001 µg mL^−1^), while it remained very low without the target virus, demonstrating that the exciton-plasmon coupling takes place only in the presence of NLP which enhances the fluorescence. Electron microscopic analysis was conducted to achieve a better insight into the bioconjugated Au NPs and carbon dots. As shown in Figure S6, the antibody-conjugated Au NPs and carbon dots led to the formation of clusters in the presence of target NLPs. From this image, it is evident that the Au NPs and carbon dots were close to each other due to the specific interaction which ultimately induced efficient fluorescence enhancement of the carbon dots by the Au NPs. Once the specificity of the present study was confirmed, differently concentrated virus solutions were added separately and monitored for changes in fluorescence emission. As shown in Fig. 5B, the emission properties of carbon dots were linearly changed at the range of 10 µg mL^−1^ to 100 pg mL^−1^ and the calculated LOD was 80.3 pg mL^−1^ based on the standard deviation method.

The specificity of the proposed sensing method was examined using bovine serum albumin (BSA), salmonella, *Escherichia coli* (*E. Coli*) and G I type NLPs. As shown in Fig. 5C, the fluorescence of carbon dots was enhanced only in the presence of target NLPs (0.001 µg mL^−1^). In the presence of BSA, salmonella, *E. Coli* and G I type NLPs, no changes were observed, which indicated the specificity of the present method toward the target virus is satisfactory.

A comparison study was performed to examine the detection sensitivity of this work with other reported articles. As shown in Table 1, the LOD value of the current study is considerable with the published articles.

**Table 1:** Comparison study of norovirus detection

Method	LOD	Ref
Colorimetric	92.7 pg mL-1	[38]
Colorimetric	80 ng mL-1	[48]
Optical fiber	1 ng mL-1	[49]
Nucleic acid sequence-based amplification	5 pg mL-1	[50]
Fluorescence	80.3 pg mL^−1^	Present work

The present sensing result was further compared with a commercial kit. As shown in Table 2, the sensitivity of the detection method was 10 times higher than the commercial norovirus detection kit. Those results showcase the superiority of the proposed sensing method and reaffirm that it would be suitable for real-life applications using handhold fluorescence spectrometers in the near future.

**Table 2:** A comparison study of the present study with the commercial kit

Detection Methods	NLP Concentration (µg mL^−1^)						
	0.00001	0.0001	0.001	0.01	0.1	1	10
Commercial Kit	**x**	**x**	**√**	**√**	**√**	**√**	**√**
Present Study	**x**	**√**	**√**	**√**	**√**	**√**	**√**

Here, √ and x denote the positive and negative detection results, respectively

## Conclusions

This study presents a new green synthesis method of carbon dots and non-spherical Au NPs. A morphological study revealed that the size of synthesized carbon dots and Au NPs were 2.5 nm and 50 nm, respectively. The proposed synthesis method of carbon dots is green and time efficient. The fluorescence emission wavelength of the carbon dots and absorbance peak of the Au NPs were located at 450 nm and 590 nm, respectively. Then, NLPs-specific antibody-conjugated carbon dots and Au NPs interacted with each other in the presence of target NLPs. The plasmon-enhanced fluorescence of carbon dots enabled the detection of NLPs with a LOD value of 80.3 pg mL^−1^. Upon comparison with a commercial kit, the present study showed 10 times more sensitivity. Most importantly, the applications of the synthesized carbon dots are not limited to fluorescence-based assay but may also offer opportunities for fluorescence imaging in the future.

## Methods

Gold (III) chloride trihydrate (HAuCl_4_·3H_2_O), bis–tris methane, N-(3-dimethylaminopropyl)-N´-ethylcarbodiimide (EDC), phosphate buffer silane (PBS), sodium formate (HCOONa), trisodium citrate and *N*-hydroxysuccinimide (NHS) were purchased from Sigma-Aldrich (St. Louis, MO, USA). Recombinant Norovirus GII.4 VP1 antigen, Recombinant Norovirus GI.3 VP1 Virus-Like Particles (strain Norovirus Hu/GI.3/JKPG_881/SWE/2007) and Mouse Anti-Norovirus GII Antibody (NP23) were received from the Native Antigen Company (KIDLINGTON, UK).

### Synthesis of fluorescent carbon dots

To synthesis fluorescent carbon dots, 2 g of citrus macroptera was added to 15 mL of MilliQ water and heated for 15 min at 100 °C. The extract of citrus macroptera was collected through filtration and kept (10 mL) into a Teflon-lined stainless-steel autoclave. Then, 100 mM Bis Tris methane (2 mL) was added to it and heated at 180 °C for 3 h. Here, citrus macroptera serves as a carbon source and buffer salt acts as a reducing & capping agent of carbon dots.

### Synthesis of gold nanoparticles

20 mL aqueous solution of HCOONa (1 mM) and HAuCl_4_ (0.0002 M) were mixed and stirred for 10 min at room temperature. Then, 0.1 mL of sodium borohydride solution a (0.5 M) was added to it and stirred for 30 min. The solution color turned purple, indicating the formation of Au NPs.

### Conjugation of carbon dots with antibodies

Hydroxyl group capped carbon dots were bound with the amino group of antibodies through electrostatic interaction. Briefly, 0.1 mL (1 µg/mL) of antibody solution (pH 5) was mixed with 0.9 mL of the carbon dots solution, and the mixture was gently stirred at 4 °C for overnight. Then, the mixture was centrifuged at 3000 rpm for 30 min to separate the conjugated antibody/carbon dots part and stored at the refrigerator before further use.

### Conjugation of Au NPs with antibodies

At first, 1 mL solution of Au NPs and EDC (4 mM) was stirred for 5 min at room temperature. Then, NHS (final conc. 10 mM) were mixed with them and incubated for 5 min. At last, 1 μL of anti-NLP Ab (final conc. 1 µg/mL) was added and stirred at 4 °C for 10 h. After centrifugation (5000 rpm for 30 min), the conjugated part was redispersed in PBS buffer (1 mL) and stored at 4 °C before further use. Then, the binding of antibodies with Au NPs was confirmed by the ELISA test before the assay experiment.

### Fluorescence detection of NLP

After confirming the binding of anti-NLP with Au NPs and QDs using the ELISA method, fluorescence sensing of NLP was performed. Shortly, anti-NLP Ab conjugated Au NPs (100 μL) containing various concentrations of NLP in PBS buffer were added into different wells of a 96 plate. Then, anti-NLP Ab-conjugated QDs (100 μL) were added to each well, and the fluorescence response of different wells was measured using fluorescence spectroscopy.

### Instrumentation

UV − visible spectroscopy and fluorescence emission wavelength were acquired by BioTek spectrophotometer (Synergy H1, USA), Horiba Lifetime Fluorometer (DeltaFlex, Tokyo, Japan) was used for Lifetime decay profile measurement, transmission electron microscopy (TEM) images of nanomaterials were taken using 80–300 LB FEI Titan (Gaithersburg, USA).

## Supplementary Information

**Additional file 1: Scheme S1.** Schematic presentation of nanomaterials synthesisand sensing method. **Figure S1.** EDX data of fluorescent carbon dots. **Figure S2.** FTIR spectra of fluorescent carbon dots. **Figure S3.** XRD spectra of fluorescent carbon dots. **Figure S4.** Zeta potential of antibodyand carbon dots. **Figure S5.** Zeta potential of antibodyand carbon dots. **Figure S6.** HR-TEM image of bioconjugated Au NPs-Carbon dots.

## Data Availability

All data generated or analyzed in this study are included in this article.
