# Supplementary material for: Non-spherical gold nanoparticles enhanced fluorescence of carbon dots for norovirus-like particles detection
Source: J Biol Eng. 2023 Apr 27;17:33. doi: 10.1186/s13036-023-00351-x (PMC10142488; doi:10.1186/s13036-023-00351-x)
Supplement: Supplementary file 1 — Additional file 1: Scheme S1. Schematic presentation of nanomaterials synthesisand sensing method. Figure S1. EDX data of fluorescent carbon dots. Figure S2. FTIR spectra of fluorescent carbon dots. Figure S3. XRD spectra of fluorescent carbon dots. Figure S4. Zeta potential of antibodyand carbon dots. Figure S5. Zeta potential of antibodyand carbon dots. Figure S6. HR-TEM image of bioconjugated Au NPs-Carbon dots. [file 13036_2023_351_MOESM1_ESM.docx]

Non-spherical Gold Nanoparticles Enhanced Fluorescence of Carbon Dots for [Norovirus-like Particles](https://www.sciencedirect.com/science/article/pii/S0956566316308594) Detection

Abdulhakeem Alzahrani^1,*^, Tawfiq Alsulami^1^, Ahmad Mohammad Salamatullah^1^, and Syed Rahin Ahmed^2^

^1^Department of Food Science & Nutrition, College of Food and Agricultural Sciences, King Saud University, Riyadh 11451, Saudi Arabia

^2^School of Engineering Practice and Technology, McMaster University, 1280 Main Street West Hamilton, Ontario, L8S 4L8, Canada

*****Correspondence: [aabdulhakeem@ksu.edu.sa](mailto:aabdulhakeem@ksu.edu.sa)


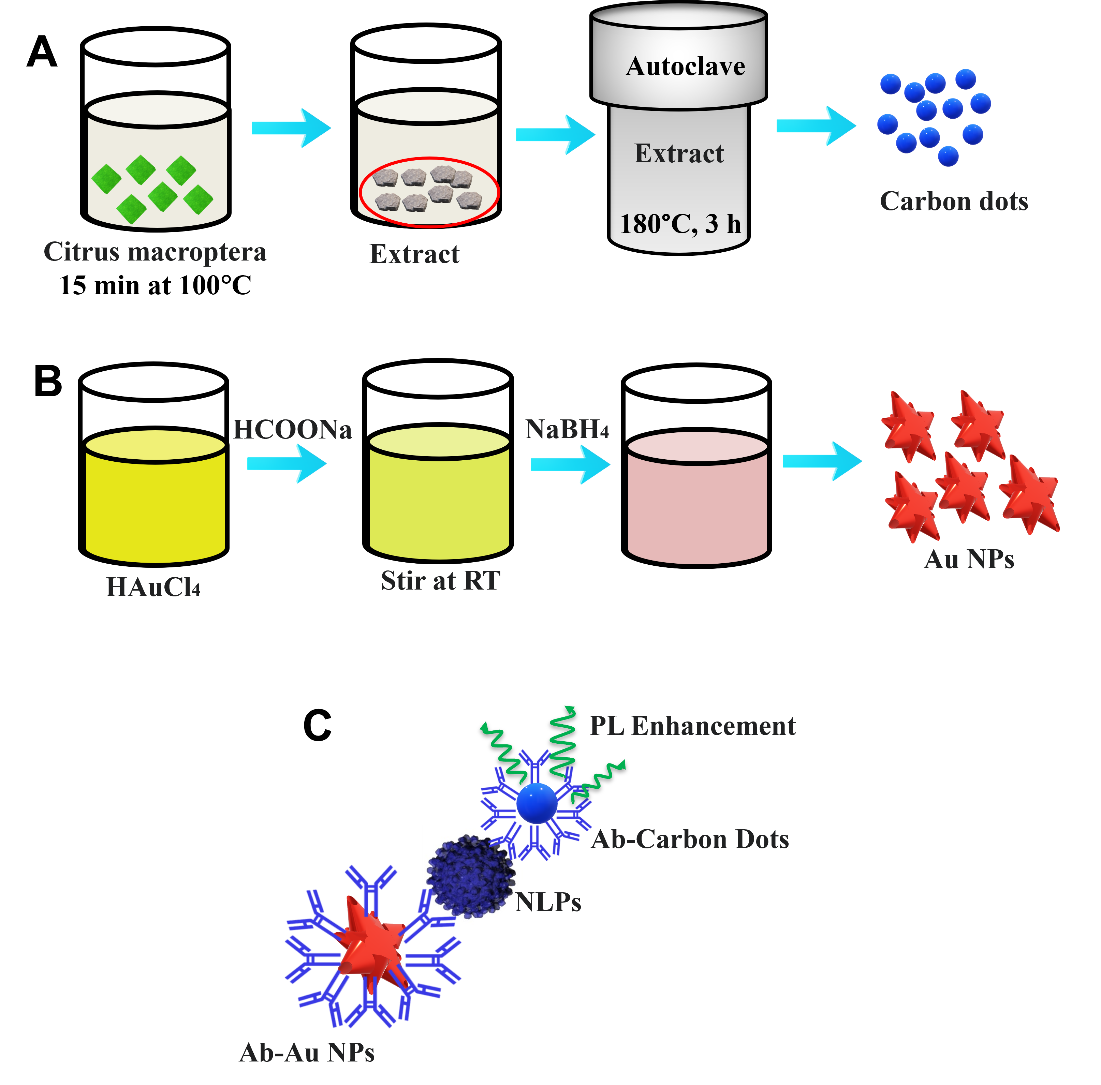


**Scheme S1:** Schematic presentation of nanomaterials synthesis (A & B) and sensing method (C).

**Characterization of Fluorescent Carbon Dots**

EDX data of synthesized nanomaterials revealed the presence of carbon in solution (Fig. S1) with negatively charge (-15.9 eV) on surface because of –OH groups (Fig. S2).


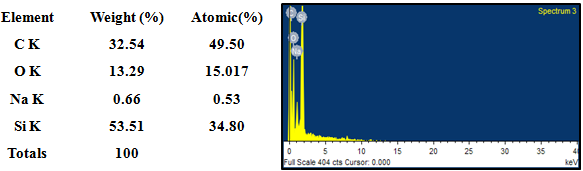


**Figure S1:** EDX data of fluorescent carbon dots.


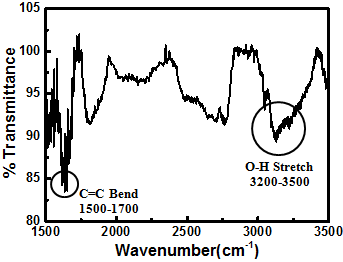


**Figure S2:** FTIR spectra of fluorescent carbon dots.


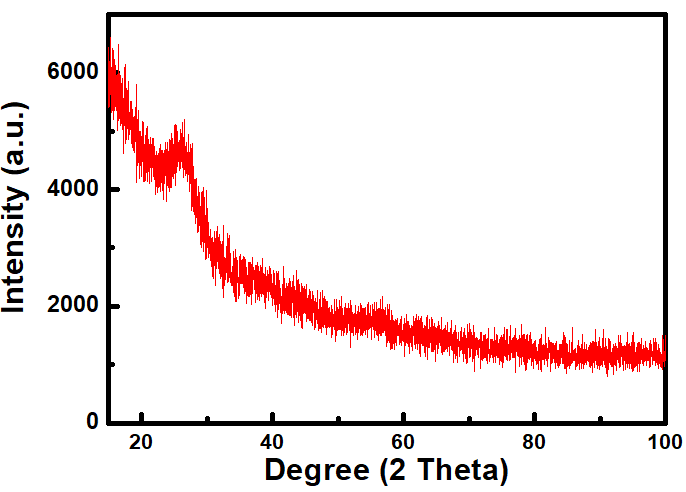


**Figure S3:** XRD spectra of fluorescent carbon dots.


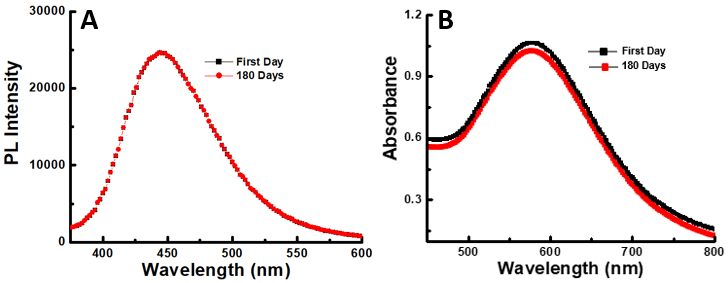


**Figure S4:** Stability of carbon dots (A) and Au NPs (B).


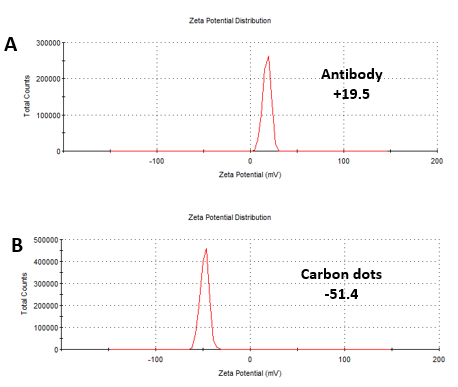


**Figure S5:** Zeta potential of antibody (A) and carbon dots (B).


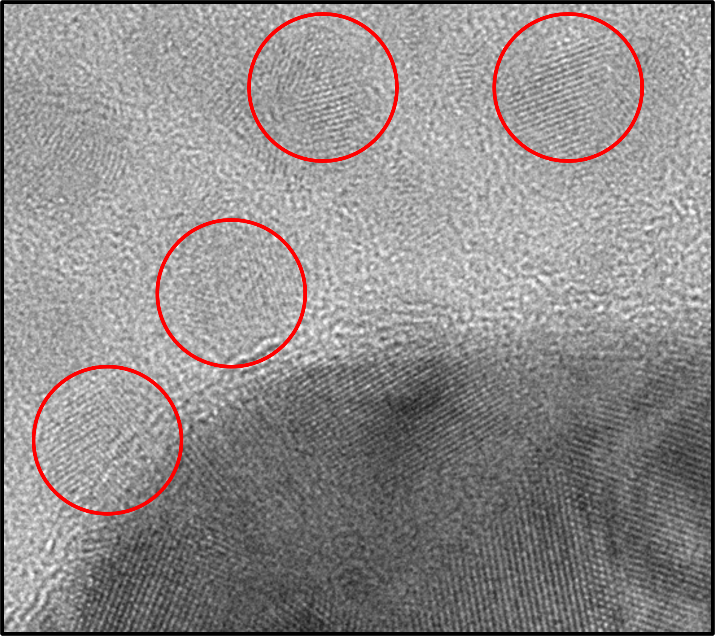


**Figure S6:** HR-TEM image of bioconjugated Au NPs-Carbon dots.
